# Intratunical injection of rat-derived bone marrow mesenchymal stem cells prevents fibrosis and is associated with increased Smad7 expression in a rat model of Peyronie’s disease

**DOI:** 10.1186/s13287-022-03090-w

**Published:** 2022-07-30

**Authors:** Wenting Wang, Weifang Ding, Xuebao Zhang, Shuang Wu, Tianxi Yu, Xin Cui, Yaqi Xie, Diandong Yang, Chunhua Lin

**Affiliations:** 1 Central Laboratory, The Affiliated Yantai Yuhuangding Hospital of Qingdao University, Yantai, 26400 China; 2 Department of Health Care, The Affiliated Yantai Yuhuangding Hospital of Qingdao University, Yantai, 26400 China; 3 Reproductive Medicine Center, The Affiliated Yantai Yuhuangding Hospital of Qingdao University, Yantai, 26400 China; 4 Department of Urology, The Affiliated Yantai Yuhuangding Hospital of Qingdao University, Yantai, 26400 China; 5 School of Clinical Medicine, Weifang Medical University, Weifang, 261000 China; 6 Binzhou Medical University, Yantai, 264000 China

**Keywords:** Bone marrow mesenchymal stem cells, Peyronie’s disease, Smad7, Osteopontin, Elastase-2B

## Abstract

**Objective:**

Peyronie’s disease (PD) is a fibrotic disorder of the penis, but effective treatments are lacking. Here, we observed the effects of rat-derived bone marrow mesenchymal stem cells (BMSCs) injection in the active phase and chronic phase in a rat model of PD, and the possible mechanism was analysed with fibroblasts derived from rat penile tunica albuginea (TA).

**Methods:**

Thirty-two male Sprague-Dawley rats were divided into four groups. In sham group, the rats were injected with 50 µL of vehicle. In the PD group, the rats were injected with 50 µg TGF-β1. In the PD + BMSCs early treatment group, the rats were injected with 50 µg TGF-β1 and injected with 1 × 10^6^ BMSCs after 1 day. In the PD + BMSCs late treatment group, the rats were injected with 50 µg TGF-β1 and injected with 1 × 10^6^ BMSCs after 28 days. Twenty-seven days after the last injection, the erectile function of the rats was measured, and then, penile fibrosis was analysed by histology and western blot. In vitro, fibroblasts derived from rat penile TA were used to identify a possible antifibrotic mechanism of BMSCs, and a Smad7 expression vector was used as a positive control. Fibroblasts were pretreated with the Smad7 expression vector or BMSCs for 48 h and then activated with 10 ng/mL TGF-β1 for 24 h. Cells viability was assessed, and Smad7, collagen 3, elastase-2B and osteopontin expression levels were analysed by immunofluorescence and western blot. Furthermore, fibroblasts were transfected with Smad7 siRNA or scramble control to observe whether the effects of BMSCs could be offset.

**Results:**

Erectile function obviously improved, and fibrosis of penile TA was prevented after BMSCs treatment compared with that in the rats with PD. Furthermore, the effects of BMSCs treatment in the active phase were better than those in the chronic phase. After cocultured with BMSCs, cell viability was not affected, Smad7 expression was upregulated, and collagen 3, elastase-2B and osteopontin levels were decreased in the TGF-β1-treated fibroblasts. After transfection with Smad7 siRNA, the antifibrotic effects of BMSCs were offset.

**Conclusions:**

The antifibrotic effects of BMSCs treatment in the active phase of the PD rat model were better than those in the chronic phase. A possible mechanism of BMSCs treatment was related to increased Smad7 expression, suggesting a possible effective and safe procedure for the treatment of PD.

**Supplementary Information:**

The online version contains supplementary material available at 10.1186/s13287-022-03090-w.

## Introduction

Tunica albuginea (TA) is mainly composed of collagen bundles and elastic fibres, surrounding the corpus cavernosum of the penis, and is essential to achieve complete rigidity during erection [1]. Peyronie’s disease (PD) is a fibrotic disorder of the penis, characterized by the formation of a fibrous plaque in the TA [2, 3]. This disease occurs in men aged 40–60 years, accompanied by penile bending and pain during sexual intercourse, and has a profound impact on patients’ relationships and quality of life [4].

Transforming growth factor-β1 (TGF-β1) plays a key role in the pathogenesis and progression of fibrosis-related diseases. Many studies have shown that TGF-β1 expression is significantly increased in penile fibrotic plaques in patients with PD [1, 3–7]. Continuous injection of exogenous TGF-β1 into rat TA at a low dose could lead to the formation of PD, and TGF-β1-specific inhibitor injection effectively reversed the fibrotic progression of TA. In an analysis of gene expression based on a DNA microarray, the genes involved in collagen synthesis, myofibroblast differentiation, tissue remodelling, inflammation, ossification, and proteolysis were found to be upregulated in the PD tissue [7, 8] and to play various roles in the progress of PD. These molecules are important indicators to determine the severity of PD. However, the exact pathophysiology of PD is still unclear, and effective treatments are lacking.

Clinically, PD is divided into an active phase and a chronic phase. In the active phase, the penis will be painful and have palpable plaques in a state of relaxation or erection. This stage will last for 12–18 months and then develop into to the chronic phase. In the chronic phase, the pain disappears and the bending of the penis is stable [9]. At present, the main treatment methods for PD include oral drugs, ultrasonic treatment, local injection of drugs in the plaque lesions and surgical correction, but the treatment effect is not ideal [9, 10]. Therefore, identification of a new treatment method that can inhibit or reverse leukoplakia fibrosis to improve PD is urgently needed.

Stem cell therapy is a noninvasive treatment method developed for the regenerative medicine, tissue engineering and cell therapeutics. Stem cells are undifferentiated cells with high plasticity that self-regenerate and differentiate. Adipose tissue-derived stem cells (ADSCs) injection was reported to prevent fibrosis and improve erectile function in a rat model of PD [1, 11]. In addition, ADSCs prevention impaired erectile function and decreased the expression of metalloproteinases (TIMPs) in a rat model of PD [4]. In a prospective study, five patients with PD were injected with placental matrix-derived mesenchymal stem cells (PM-MSCs), and statistically significant increases in peak systolic velocity occurred after PM-MSC injection [12]. Surface marker characteristics are similar between bone marrow mesenchymal stem cells (BMSCs) and ADSCs, but there are various differences with regard to the mechanisms involved in tissue repair, immune regulation and haematopoiesis support [13–15]. BMSCs showed a higher chondrogenic capacity than ADSCs [15]. The fibroblasts could be directly reprogrammed into chondrocytes using a 3D system with a chemical cocktail [16]. Therefore, BMSCs might be a more effective treatment method for PD. Nevertheless, the effects of BMSCs on different phases of PD and their possible mechanisms have not been reported. A systematic study of BMSCs in PD is needed.

In this study, the effects of rat BMSCs injection at the active phase and chronic phase in a rat model of PD were observed, and the possible mechanism was analysed using fibroblasts derived from rat penile TA.

## Materials and methods

### Animals and BMSCs

Thirty-two male Sprague-Dawley rats (11–12 weeks old, 300–350 g) from Ji’nan Pengyue Laboratory Animal Breeding Co., Ltd. (Shandong, China, Approval No.: SCXL (Lu)2019003) were used in this study. All animals were housed in a standard experimental laboratory with freely available food under 12 h reversed cycle light.

The rat BMSCs (RASMX-01001, OriCell Sprague–Dawley) were purchased from Cyagen (Jiangsu, China) and cultured with its special complete medium (RASMX-90011, Cyagen) at 37 ℃ and 5% CO_2_. The fluid change interval was 2–3 days. When the cells reached 80–90% confluence, they were used for experiments.

### Animal groups

Before surgery, rats were anaesthetized with pentobarbital sodium (45 mg/kg). Based on the existing literature [1, 4], the rat model of PD was established by injection of 50 µg TGF-β1 in the right midshaft dorsomedial TA with a microlitre syringe. All rats were randomly divided into four groups, with 8 in each group.

In the sham group, 50 µL of phosphate-buffered saline (PBS) was injected into the right midshaft dorsomedial TA, followed by a second injection with 50 µL of PBS after 1 day and a third injection with 50 µL of PBS after 27 days.

In the PD group, 50 µg of TGF-β1 in 50 µL PBS was injected into the TA, followed by a second injection with 50 µL of PBS after 1 day and a third injection with 50 µL of PBS after 27 days.

In the BMSCs early treatment group, 50 µg of TGF-β1 was injected into TA, followed by injection with 1 × 10^6^ BMSCs in 50 µL of PBS after 1 day and a third injection with 50 µL of PBS after 27 days.

In the BMSCs late treatment group, 50 µg of TGF-β1 was injected into TA, followed by injection with PBS after 1 day, and 1 × 10^6^ BMSCs in 50 µL of PBS were injected into the TA after 27 days.

**Fig. 1:**
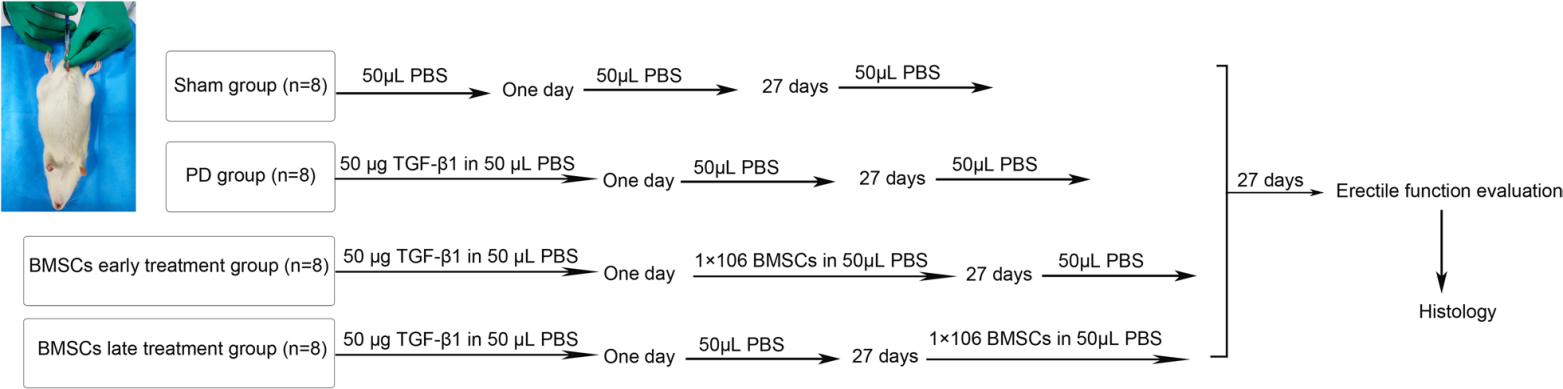
The experimental design of the animals study

Twenty-seven days after the third treatment, the rats were subjected to erectile function evaluation in each group and killed to obtain the penises. The experimental design is shown in Fig. 1.

### Erectile function evaluation

According to previous reports [1, 4], erectile function was evaluated by the intracavernous pressure (ICP) response to electrostimulation of the cavernous nerve (CN). Briefly, under anaesthesia with pentobarbital sodium, the right crus of the corpus cavernosum was cannulated with a heparinized (200 U/mL) 25-G needle connected to a pressure transducer. The right CN was activated (2.5, 5, and 7.5 V) by platinum electrodes connected to a stimulator at 20 Hz for 50 s. Mean arterial pressure (MAP) was recorded by carotid artery cannulation. A rest period of at least 3 min was allowed for nerve recovery between CN stimulation trials.

### Haematoxylin & eosin (HE) and Masson staining

After erectile function evaluation, the penis was collected and fixed with 4% paraformaldehyde. After serial coronal sections (3 µm) were generated, the sections were deparaffinized with xylene and rehydrated with ethanol (100%, 5 min; 95%, 2 min; 80%, 2 min; 70%, 2 min). For HE staining, the sections were stained with haematoxylin for 5 min and eosin for 15 s. For Masson staining, the sections were stained according to the Masson’s trichrome-staining kit. The results were observed and imaged with a light microscope.

### Immunohistochemistry

After three washes with 0.01 mol/L PBS, the penis slices were cultured with 3% H_2_O_2_ (10 min). The primary antibodies against collagen 3 (1:200, AF5457, Affinity, China), Smad7 (1:200, AF5147, Affinity, China), elastase-2B (1:200, orb471854, Biorbyt, China), and osteopontin (1:200, AF0227, Affinity, China) were cultured with slices overnight at 4 °C, respectively. After PBS washes, the goat anti-rabbit IgG (H + L) secondary antibody (1:5000, #S0001, Affinity, China) was cultured with slices at 37 °C for 60 min. After staining with diaminobenzidine (DAB), the slices were dehydrated, purified and sealed with neutral gum. The results were observed and photographed with a light microscope.

### Fibroblast culture and transfection

Fibroblasts derived from rat penile TA were purchased from Procell (CP-R316, Wuhan, China). The fibroblasts were cultured with special complete culture medium (CM-R316, Procell) at 37 ℃ and 5% CO_2_. The culture medium was changed after 24 h, and then, the medium was changed once every 3 days. When the cells reached 80–90% confluence, they were used for experiments.

According to a previous report [2], fibroblasts were transfected with 1 µg PEI25k/pCMV5-Smad7 vector after serum-starvation for 24 h. In parallel, an empty PEI25k/pCMV5 vector was transfected as a control. The pCMV5-Smad7 gene (mouse GenBank ID: AF015260.1) was purchased from Addgene (Cambridge, MA, USA). The PEI25k/pCMV5-Smad7 vector was prepared by transient transfections using hyperbranched poly (PEI25k, Sigma-Aldrich). After transfection, the fibroblasts were cultured for 48 h.

According to a previous study [17], fibroblasts were transfected with 40 nM Smad7 small interfering RNA (siRNA, Invitrogen) or scramble control siRNA for 48 h with Lipofectamine 3000 reagent.

### Coculture of fibroblasts and BMSCs

The fibroblasts were cocultured with BMSCs using 6-well Transwell plates. In detail, fibroblasts (5 × 10^4^ cells/well in 2 mL) were added to the upper chamber, and BMSCs (2 × 10^5^ cells/well in 2 mL) were added to the lower chamber. Following indirect coculture for 48 h, fibroblasts were collected.

### Cell groups

#### Design 1

The fibroblasts were randomly divided into five groups. In the control group, the cells were cultured under normal conditions without any treatment. In the TGF-β1 group, the cells were treated with 10 ng/mL TGF-β1 for 24 h. In the TGF-β1 + vector group, the cells were transfected with empty PEI25k/pCMV5 vector for 48 h and then treated with 10 ng/mL TGF-β1 for 24 h. In the TGF-β1 + pSmad7 group, the cells were transfected with PEI25k/pCMV5-Smad7 vector (pSmad7) for 48 h and then treated with 10 ng/mL TGF-β1 for 24 h. In the TGF-β1 + BMSCs group, the cells were cocultured with BSMCs for 48 h and treated with 10 ng/mL TGF-β1 for 24 h.

#### Design 2

To confirm that Smad7 is the key factor mediating the antifibrotic effects of BMSCs, we transfected fibroblasts with Smad7 siRNA or scramble control to determine whether the effects of BMSCs could be offset. The cells were divided into the following four groups: scramble group, Smad7 siRNA group, scramble + BMSCs group and Smad7 siRNA + BMSCs group. In the scramble or Smad7 siRNA group, the cells were transfected with 40 nM scramble or Smad7 siRNA for 48 h and treated with 10 ng/mL TGF-β1 for 24 h. In the scramble + BMSCs or Smad7 siRNA + BMSCs group, the transfected cells were cocultured with BSMCs for 48 h and treated with 10 ng/mL TGF-β1 for 24 h.

### Cell viability

Fibroblasts viability was determined by a CCK-8 kit and cell migration experiments. According to a standard protocol, 10 µL of CCK-8 solution was added to each well for 4 h. The absorbance of each well was measured by a microplate reader at 450 nm. Fibroblasts (5 × 10^5^ cells/mL) were added to the upper chamber of Transwell plates, and special complete culture medium was added to the lower chambers. After incubation for 48 h, the fibroblasts were fixed, stained and washed according to a standard protocol.

### Immunofluorescence

Fibroblasts were fixed with 4% paraformaldehyde and washed as a standard protocol. After the cells were blocked with TBS-T containing 5% BSA for 60 min, they were incubated with antibodies against Smad7 (1:200, AF5147, Affinity, China), collagen3 (1:200, AF5457, Affinity, China), elastase-2B (1:200, orb471854, Biorbyt, China), and osteopontin (1:200, AF0227, Affinity, China) overnight at 4 ℃. Washing three washes with TBS-T, the fluorescein isothiocyanate-conjugated solution was added and incubated for 60 min. The nuclei of the cells were stained with 4′,6-diamidino-2-phenylindole (DAPI). A confocal microscope was used to observe the results.

### Western blot

Based on a previously reported protocol [1], the dorsal neurovascular bundle and buck fascia were removed from the TA and urethra. The tissues and fibroblasts in each group were lysed in RIPA buffer (R0010, Solarbio, China). Twelve per cent SDS-PAGE was used to separate proteins (30 μg), and the separated proteins were transferred to PVDF membranes (EMD Millipore). Appropriate primary antibodies diluted with 5% BSA were incubated with the membranes overnight at 4 °C. The primary antibodies consisted of collagen 3 (1:800, AF5457, Affinity, China), Smad7 (1: 800, AF5147, Affinity, China), elastase-2B (1: 800, orb471854, Biorbyt, China), osteopontin (1: 800, AF0227, Affinity, China) and GAPDH (1:1000, BA2913, Boster, China). After 3 washes with TBS-0.01% Tween 20, the HRP-Affinipure goat anti-rabbit IgG (H + L) (1:500, BM3894, Boster, China) was incultured with the membranes for 2 h at 25 °C. After washing, the signals were visualized using enhanced chemiluminescence reagent (D085075, Bio-Rad, China).

### Statistical analysis

All data were analysed with SPSS 19.0 software (IBM SPSS, Chicago, IL, USA) and are expressed as the mean ± standard deviation. One-way analysis of variance was used to compare differences among groups, followed by Tukey’s post. *P* < 0.05 was considered significant.

## Results

### BMSCs injection improved erectile function and prevented fibrosis in a rat model of PD

**Fig. 2:**
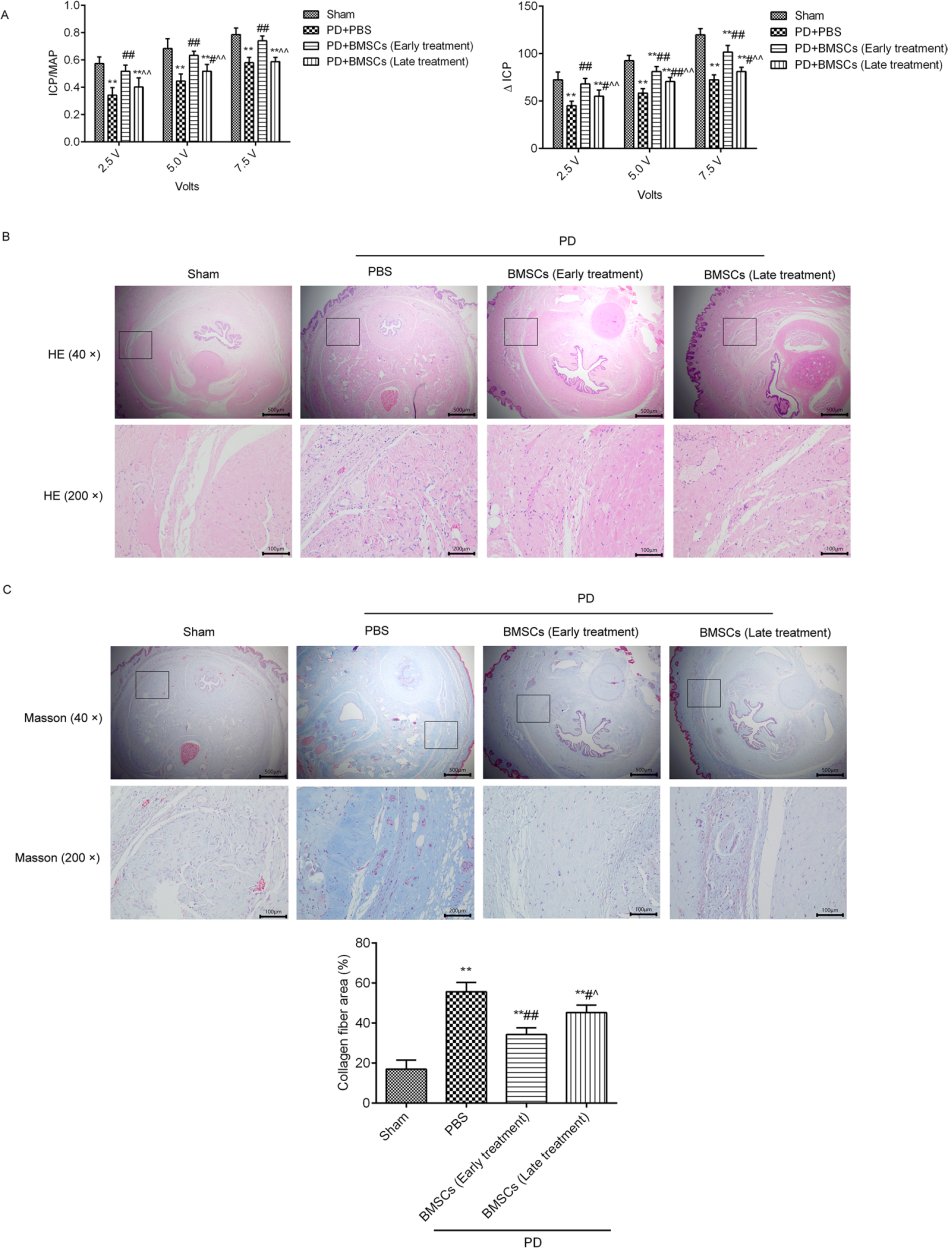
BMSCs injection improved erectile function and prevented fibrosis in a rat model of PD. **A** Erectile function measurement in each group at various voltages during cavernous nerve electrostimulation. ICP/MAP: intracavernous pressure (ICP) normalized over mean arterial pressure (MAP); ∆ ICP: ICP change from baseline to peak ICP. **B** Histological changes in penile tissue cross sections were observed by H&E. BMSCs (early treatment): BMSCs were treated at the active phase of PD; BMSCs (late treatment): BMSCs were treated at the chronic phase of PD. **C** Fibrotic changes in penile tissue cross sections were observed by Masson. Collagen fibre area was analysed using ImageJ software (version 6.1, USA). ^**^P < 0.01 versus the sham group; ^#^*P* < 0.05, ^##^*P* < 0.01 versus the PD + PBS group; ^^^*P* < 0.05, ^^^^*P* < 0.01 the versus PD + BMSCs (early treatment) group

As shown in Fig. 2A, we found a significantly diminished response to cavernous nerve electrostimulation (the levels of ICP/MAP and ∆ICP were decreased) in the PD group when compared to the sham group. In active phase of PD, intratunical injection of BMSCs (early treatment) strongly increased the response to cavernous nerve electrostimulation by increasing the ICP/MAP and ∆ICP. In chronic phase of PD, intratunical injection of BMSCs (late treatment) increased the response to cavernous nerve electrostimulation by increasing the ICP/MAP and ∆ICP values. However, the effects of early BMSCs treatment were better than those of late BMSCs treatment.

The results of H&E staining (Fig. 2B) showed an increased number of cells in the TA of the PD group, which significantly increased the density of TA. After treatment with BMSCs at different phases of PD, the cells numbers were obviously decreased in the TA. Through Masson staining (Fig. 2C), we found that TGF-β1 injection induced collagen fibres in the TA, and the degree of collagen fibres in the TA was suppressed by BMSCs treatment. Notably, BMSCs treatment in the active phase of PD showed stronger inhibition of the collagen fibres of TA than treatment in the chronic phase.

### BMSCs injection inhibited collagen 3 expression and promoted Smad7 expression in the TA of rats with PD

**Fig. 3:**
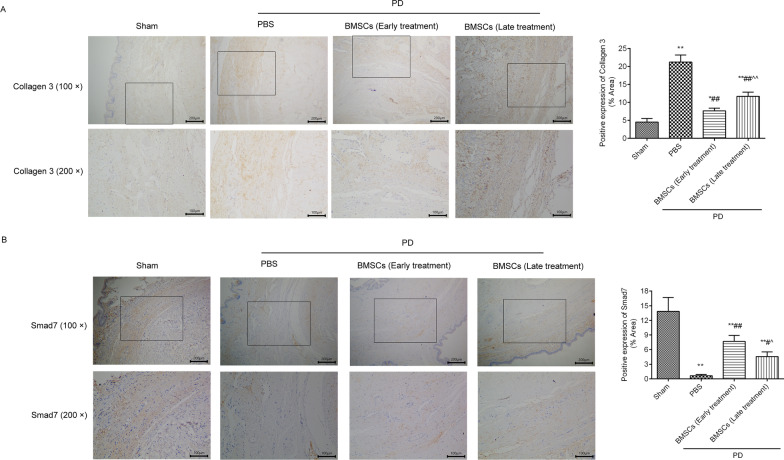
BMSCs injection prevented collagen 3 expression and promoted Smad7 expression in the TA of the rats with PD. The positive expression levels of collagen 3 **A** and Smad7 **B** were analysed by immunohistochemistry. The positive expression levels were analysed using ImageJ software. ^*^P < 0.05, ^**^P < 0.01 versus the sham group; ^#^*P* < 0.05, ^##^*P* < 0.01 versus the PD + PBS group; ^^^*P* < 0.05, ^^^^*P* < 0.01 versus the PD + BMSCs (early treatment) group

The expression of collagen 3 (Fig. 3A) and Smad7 (Fig. 3B) in the TA was assessed by immunohistochemistry. As shown in Fig. 3A, the positive expression of collagen 3 in the TA was significantly increased in the PD group contrasted to the sham group. BMSCs treatment clearly declined the expression of collagen 3 compared with that in the PD group, and the effects of BMSCs early treatment were better than those of late treatment. In Fig. 3B, the positive expression of Smad7 in the TA was clearly downregulated in the PD group compared to the sham group. After treatment with BMSCs, the expression levels of Smad7 were upregulated in the TA. Additionally, BMSCs treatment in the active phase increased Smad7 expression more than BMSCs treatment in the chronic phase. The western blot results (Fig. 5, full-length gels were included in Additional file 1) were similar to the immunohistochemistry results: collagen 3 expression significantly increased in the rat with PD and decreased after BMSCs treatment (Fig. 5C). Smad7 expression significantly decreased in the rats with PD and increased after BMSCs treatment (Fig. 5B). The effects of BMSCs treatment in the active phase were better than those in the chronic phase.

### BMSCs injection suppressed elastase-2B and osteopontin in the TA of rats with PD

**Fig. 4:**
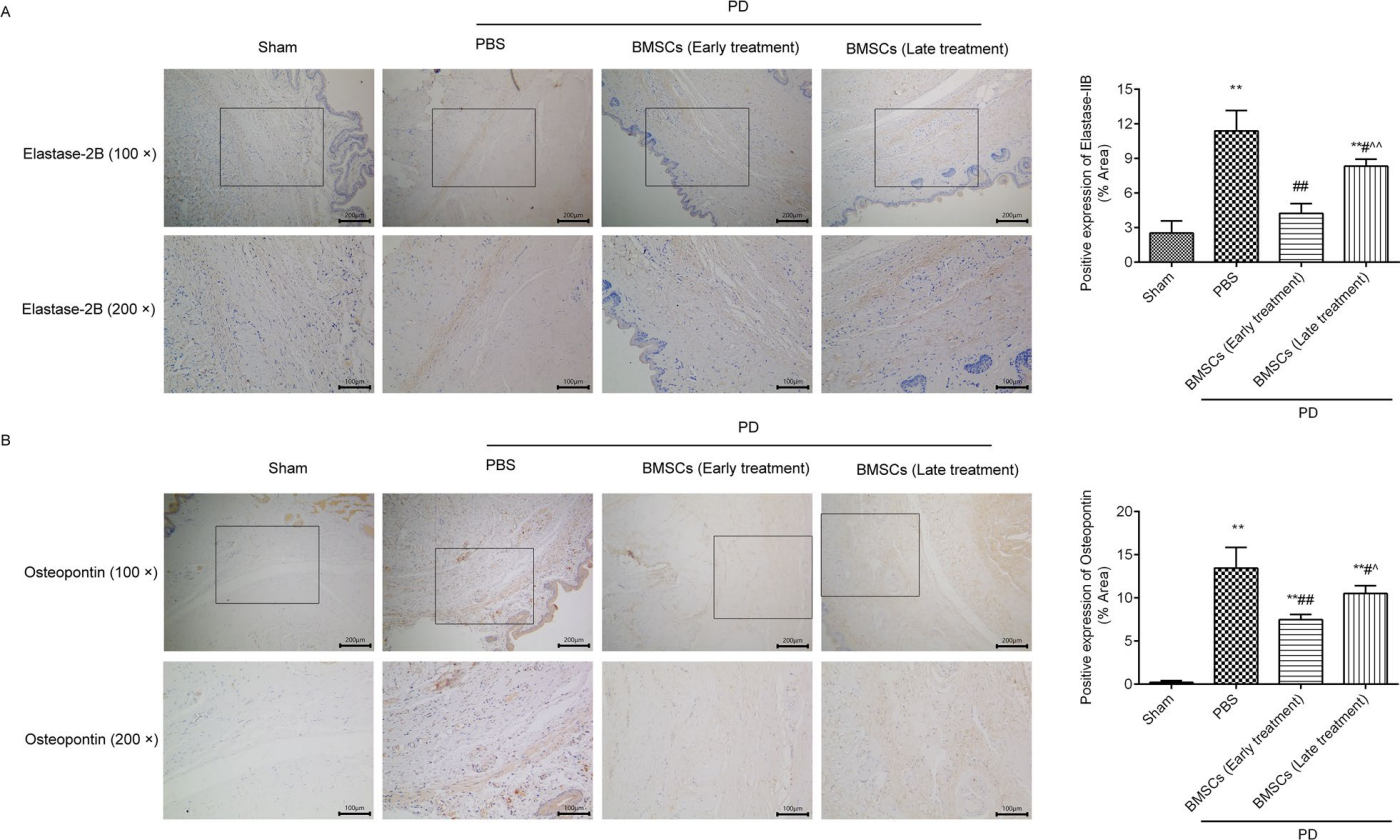
BMSCs injection prevented elastase-2B and osteopontin in the TA of the rats with PD. The positive expression levels of elastase-2B **A** and osteopontin **B** were analysed by immunohistochemistry. The positive expression levels were analysed using ImageJ software. ^*^P < 0.05, ^**^P < 0.01 versus the sham group; ^#^*P* < 0.05, ^##^*P* < 0.01 versus the PD + PBS group; ^^^*P* < 0.05, ^^^^*P* < 0.01 versus the PD + BMSCs (early treatment) group

**Fig. 5:**
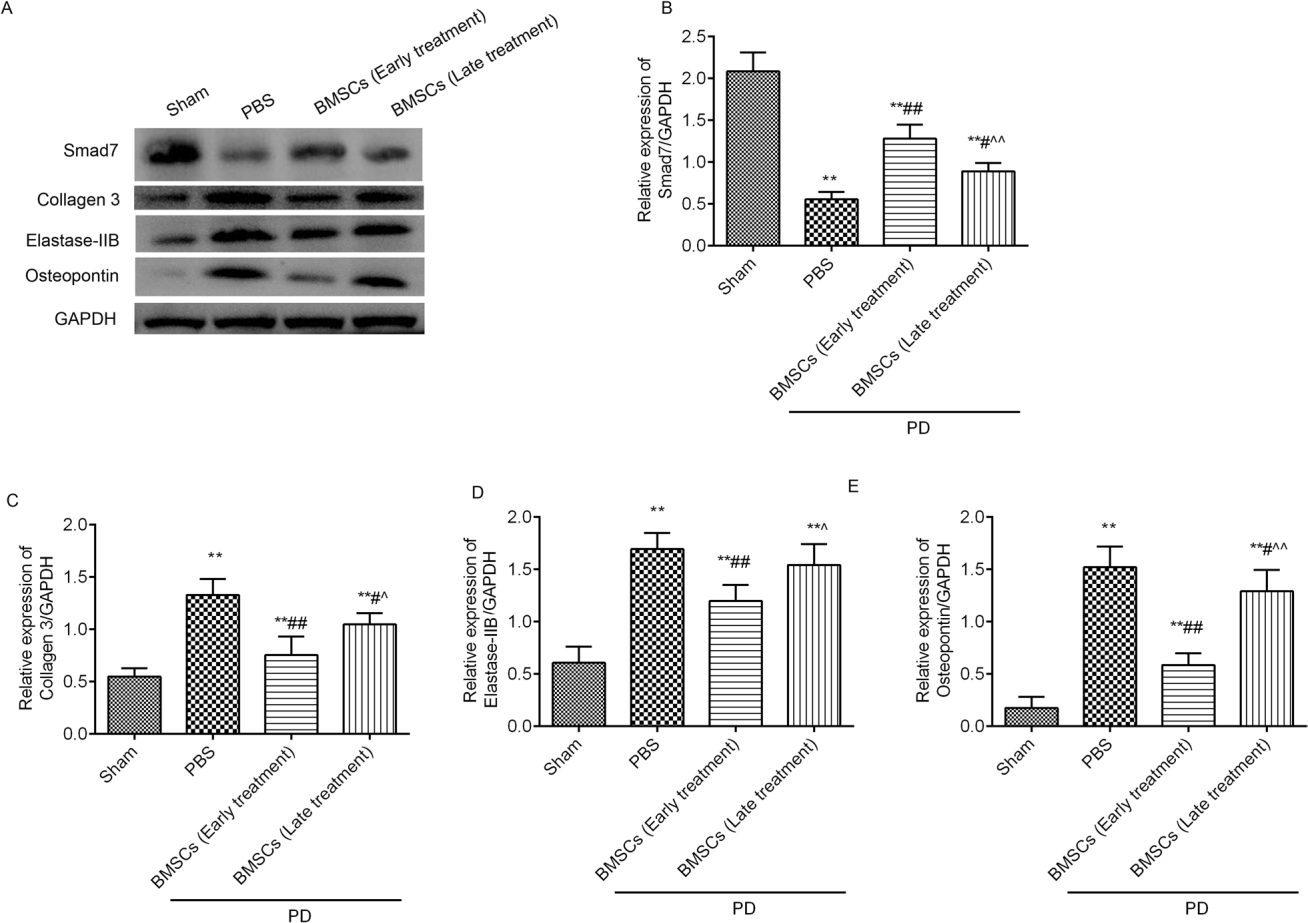
The expression levels of Smad7, collagen 3, elastase-2B and osteopontin in the TA of the rats with PD were analysed by western blot (**A**). The full-length gels were included in Additional file 1. The relative expression levels of Smad7 (**B**), collagen 3 (**C**), elastase-2B **D** and osteopontin **E** were analysed by ImageJ software. ^*^P < 0.05, ^**^P < 0.01 versus the sham group; ^#^*P* < 0.05, ^##^*P* < 0.01 versus the PD + PBS group; ^^^*P* < 0.05, ^^^^*P* < 0.01 versus the PD + BMSCs (early treatment) group

Positive expression of elastase-2B (Fig. 4A) and osteopontin (Fig. 4B) was found in the TA by immunohistochemistry. The positive expression of elastase-2B and osteopontin in the TA was significantly increased in the PD group when contrasted to the sham group. BMSCs treatment clearly declined the expression of elastase-2B and osteopontin compared with that of the PD group, and the effects of BMSCs early treatment were better than those of late treatment. The western blot results (Fig. 5, full-length gels were included in Additional file 1) also showed that elastase-2B (Fig. 5D) and osteopontin (Fig. 5E) levels were significantly increased in the rats with PD, and BMSCs treatment decreased their expression levels.

### Smad7 expression increased in TGF-β1-treated fibroblasts after coculture with BMSCs

**Fig. 6:**
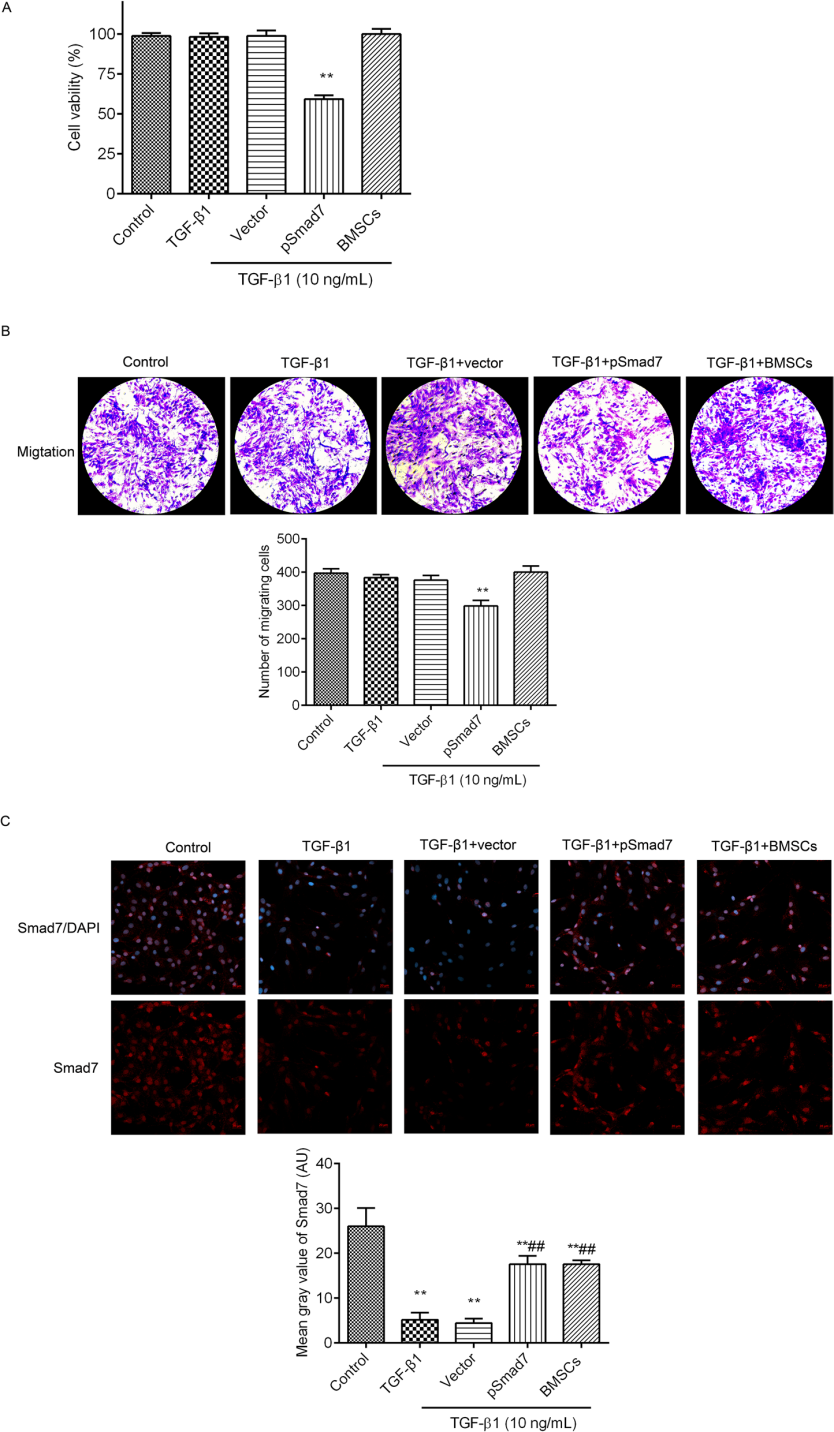
Smad7 expression increased in the TGF-β1-induced fibroblasts after coculture with BMSCs. **A** The cell viabilities of fibroblasts were analysed by CCK-8. **B** The migration of fibroblasts was analysed by Transwell plates (scale = 180 µm). **C** Smad7 expression was observed by immunofluorescence (scale = 20 µm). The numbers of migrating cells and the mean grey value of Smad7 were analysed by ImageJ software. ^**^P < 0.01 versus the control group; ^##^*P* < 0.01 versus the TGF-β1 + vector group

To confirm that the mechanism of BMSCs treatment is related to Smad7 in the TA of the rats with PD, we transfected the Smad7 expression vector into the fibroblasts as a positive control. Cells activity (Fig. 6A) and migration (Fig. 6B) were measured, and the results showed that Smad7 overexpression suppressed cells activity and migration in the TGF-β1-treated fibroblasts. However, cells activity and migration were not affected in the TGF-β1-treated fibroblasts, after coculture with BMSCs. The expression levels of Smad7 were observed by immunofluorescence (Fig. 6C) and western blot (Fig. 8B, full-length gels were included in Additional file 2). In contrast to those in the control group, Smad7 expression levels were decreased in the TGF-β1-treated fibroblasts. The Smad7 expression vector or coculture with BMSCs obviously enhanced the expression levels of Smad7 in the TGF-β1-treated fibroblasts.

### Collagen 3, elastase-2B and osteopontin expression levels were suppressed in TGF-β1-treated fibroblasts after coculture with BMSCs

**Fig. 7:**
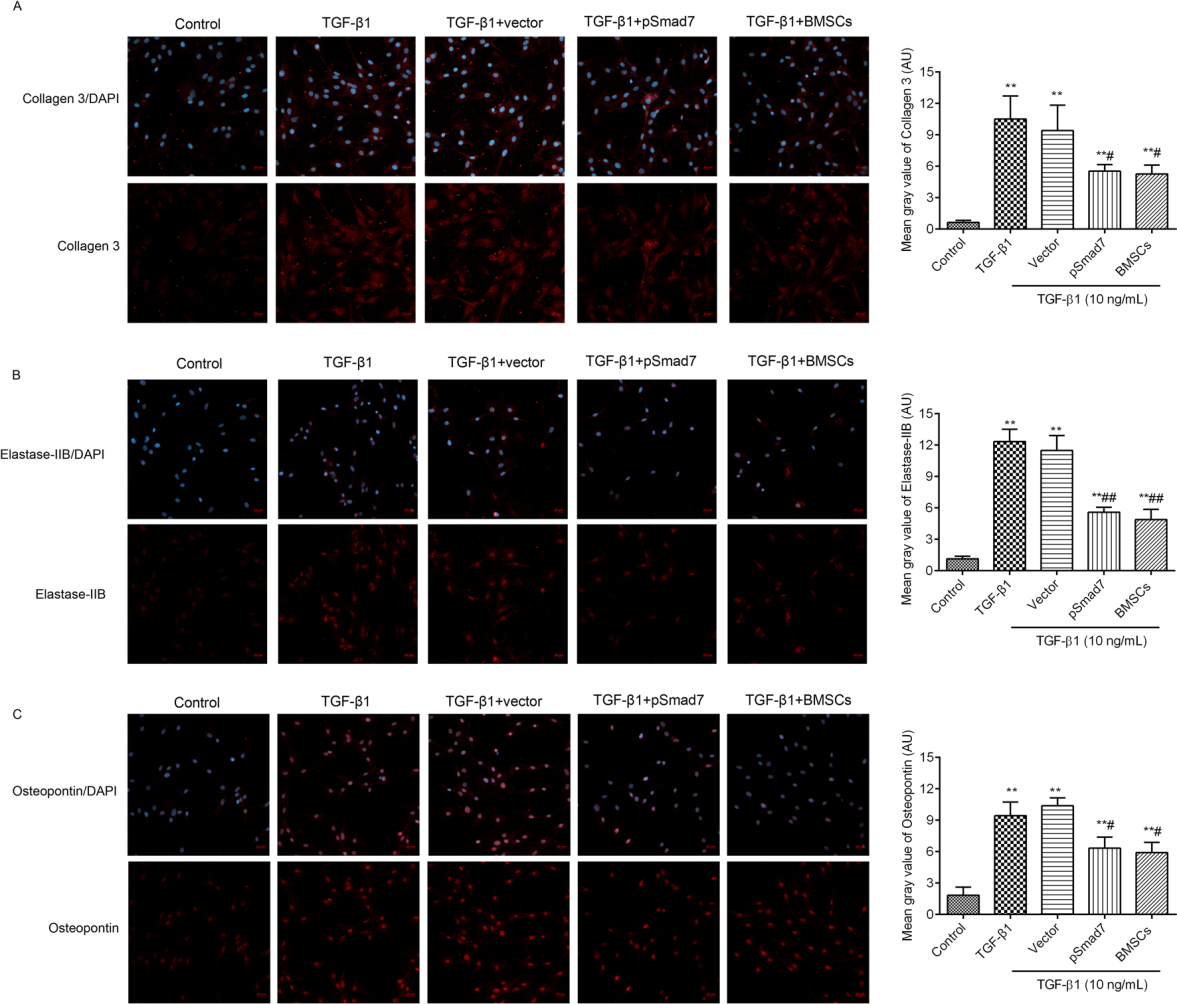
Collagen 3, elastase-2B and osteopontin expression levels decreased in the TGF-β1-induced fibroblasts after coculture with BMSCs. Collagen 3 **A**, elastase-2B **B** and osteopontin **C** expression was observed by immunofluorescence (scale = 20 µm). The mean grey values were analysed by ImageJ software. ^**^P < 0.01 versus the control group; ^#^*P* < 0.05, ^##^*P* < 0.01 versus the TGF-β1 + vector group

**Fig. 8:**
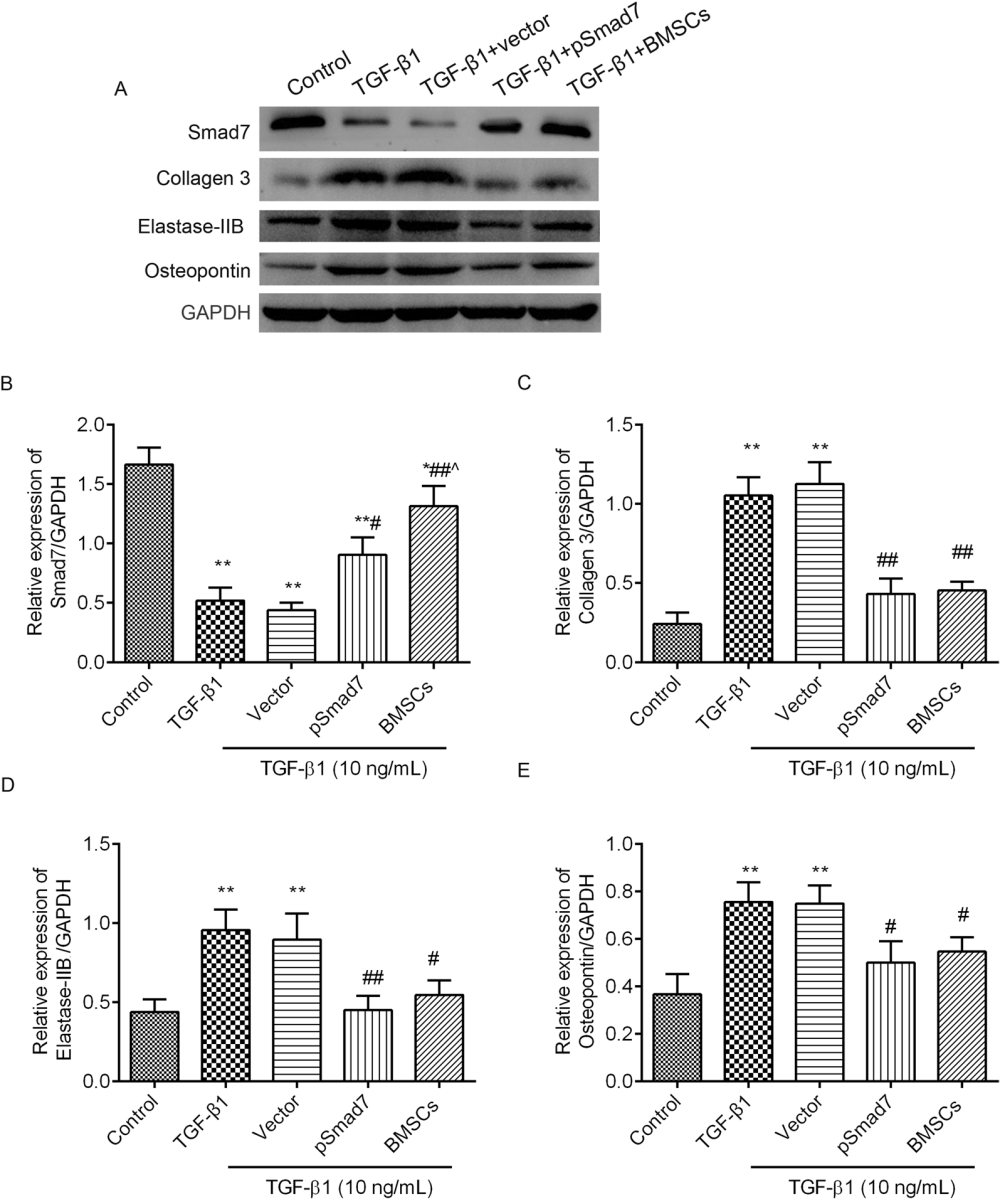
Smad7, collagen 3, elastase-2B and osteopontin expression levels in the TGF-β1-induced fibroblasts after coculture with BMSCs by western blot **A**. The full-length gels were included in Additional file 2. The relative expression of Smad7 **B**, collagen 3 **C**, elastase-2B **D** and osteopontin **E** expression was analysed by ImageJ software. ^**^P < 0.01 versus the control group; ^#^*P* < 0.05, ^##^*P* < 0.01 versus the TGF-β1 + vector group; ^*P* < 0.05 versus the pSmad7 group

The expression levels of collagen 3, elastase-2B and osteopontin in the fibroblasts were measured by immunofluorescence (Fig. 7) and western blot (Fig. 8, full-length gels were included in Additional file 2). In the TGF-β1-treated fibroblasts, collagen 3 (Figs. 7A and 8C), elastase-2B (Figs. 7B and 8D) and osteopontin (Figs. 7C and 8E) expression levels were increased compared to those in the control cells. A Smad7 expression vector or coculture with BMSCs obviously prevented the expression of the three proteins in the TGF-β1-treated fibroblasts.

### Inhibition of Smad7 offset the antifibrotic effects of BMSCs in TGF-β1-treated fibroblasts

**Fig. 9:**
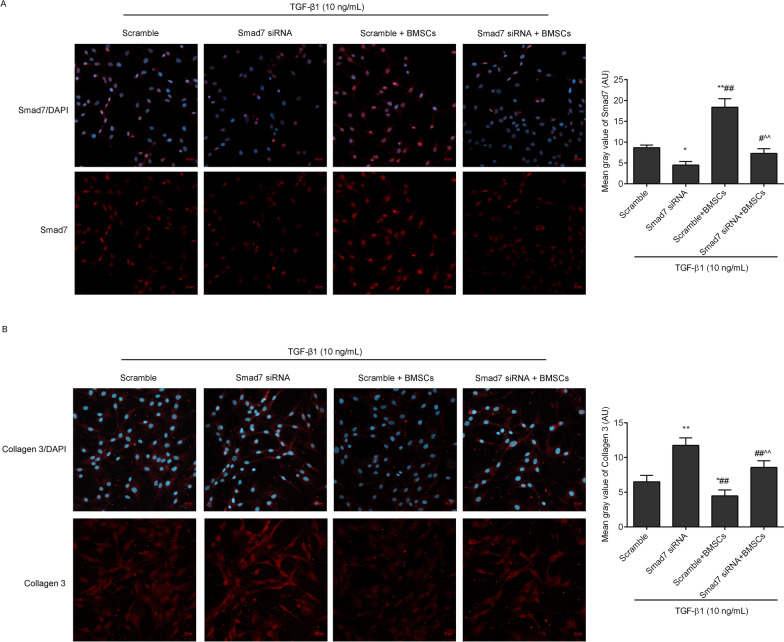
Smad7 and collagen 3 expression levels were observed in TGF-β1-induced fibroblasts by immunofluorescence (scale = 20 µm). The mean grey values were analysed by ImageJ software. ^*^P < 0.05, ^**^P < 0.01 versus the scramble group; ^#^*P* < 0.05, ^##^*P* < 0.01 versus the Smad7 siRNA group; ^^^^*P* < 0.01 versus the scramble + BMSCs group

**Fig. 10:**
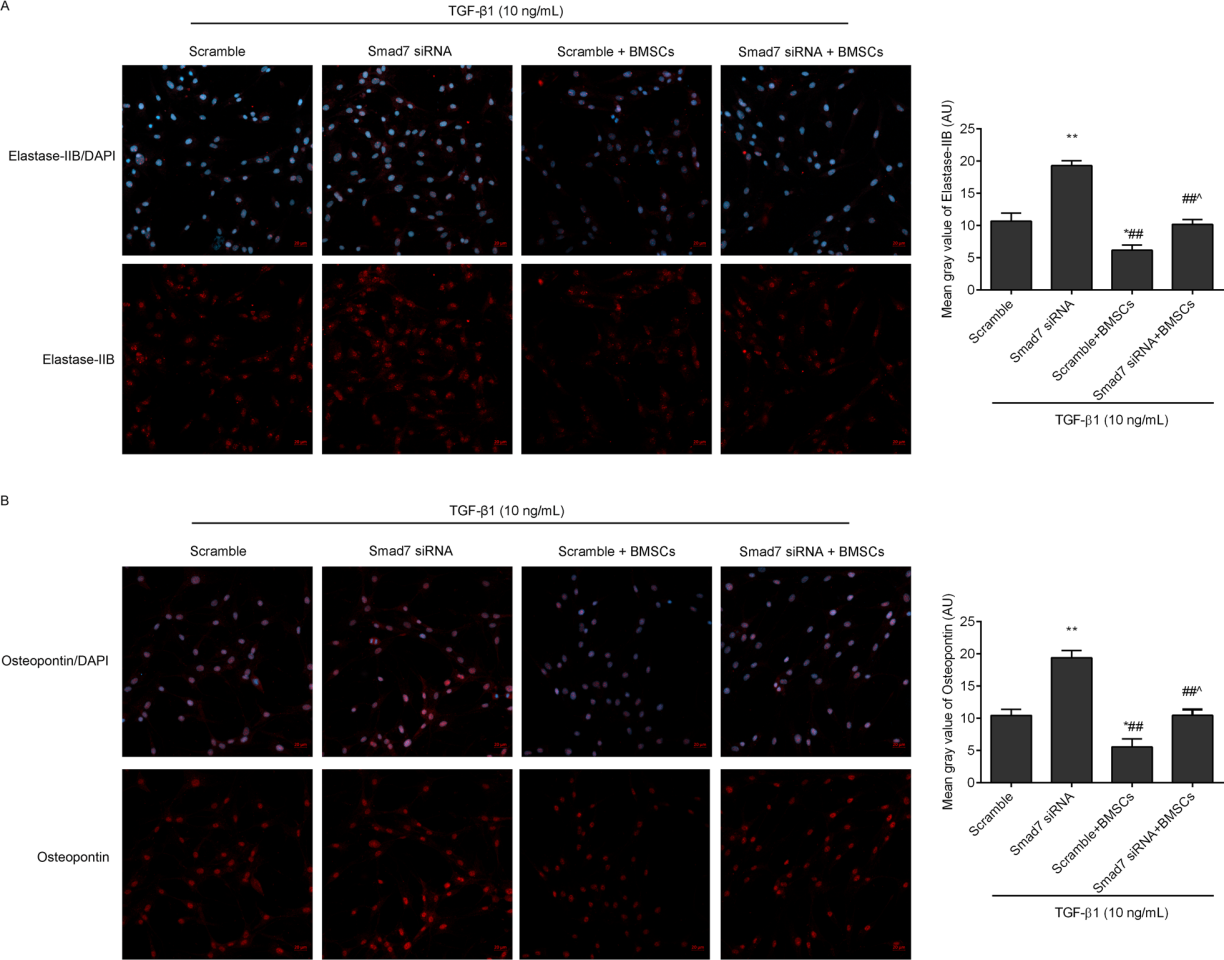
Elastase-2B and osteopontin expression levels were observed in the TGF-β1 induced fibroblasts by immunofluorescence (scale = 20 µm). ^*^P < 0.05, ^**^P < 0.01 versus scramble group; ^##^*P* < 0.01 versus Smad7 siRNA group; ^^^*P* < 0.05 versus scramble + BMSCs group

**Fig. 11:**
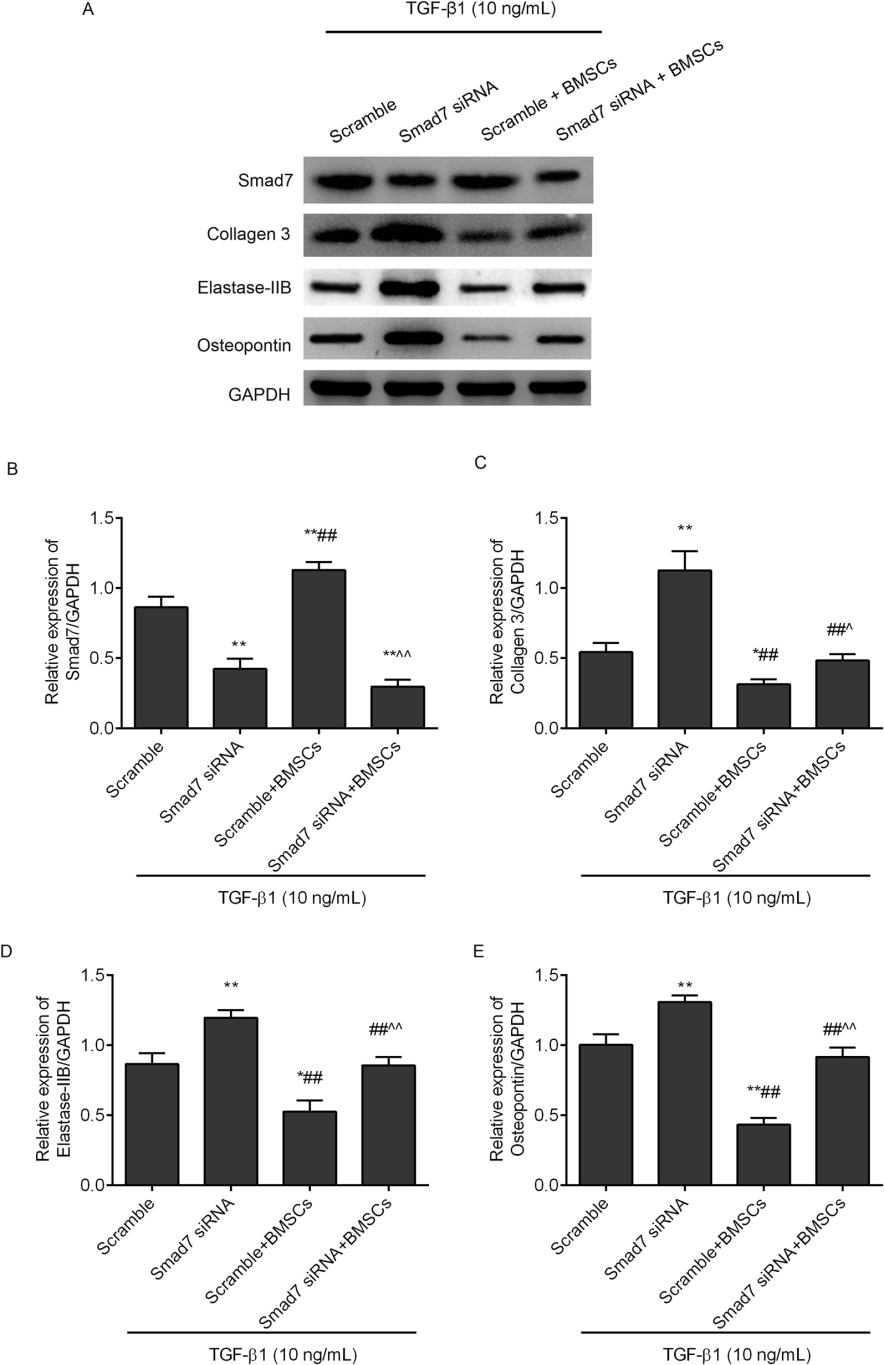
Smad7, collagen 3, elastase-2B and osteopontin expression levels were observed in the TGF-β1-induced fibroblasts by western blot. The full-length gels were included in Additional file 3. **A** The proteins bands; The relative expression of Smad7 (**B**), collagen 3 (**C**), elastase-2B **D** and osteopontin **E** expression was analysed by ImageJ software. ^*^P < 0.05, ^**^P < 0.01 versus the scramble group; ^##^*P* < 0.01 versus the Smad7 siRNA group; ^^^*P* < 0.05, ^^^^*P* < 0.01 versus the scramble + BMSCs group

In order to confirm Smad7 was the key factor mediating antifibrotic effects of BMSCs, we transfected fibroblasts with Smad7 siRNA to observe the effects of BMSCs. As shown in Figs. 9A and 11B (full-length gels were included in Additional file 3), the Smad7 expression levels were downregulated by Smad7 siRNA in the TGF-β1 induced fibroblasts compared with the scramble group. After coculture with BMSCs, the upregulation of Smad7 in BMSCs was hampered by Smad7 siRNA transfection. Furthermore, we observed the expression levels of collagen 3 (Figs. 9B and 11C), elastase-2B (Figs. 10A and 11D) and osteopontin (Figs. 10B and 11E) in fibroblasts. The data showed that inhibition of Smad7 upregulated collagen3, elastase-2B and osteopontin in the TGF-β1-treated fibroblasts. Meanwhile, the anti-fibrosis effects of BMSCs in the TGF-β1-treated fibroblasts were blocked by Smad7 siRNA transfection.

## Discussion

There are many hypotheses about the pathogenesis of PD, such as chronic trauma, impaired fibrin clearance, autoimmunity and genetic pathogenesis [18]. Studies have suggested that the repeated injury of penile white membrane microvessels leads to the extravasation of fibrin in the wound, and fibrin promotes TGF-β1 expression release and causes the transdifferentiation of fibroblasts into myofibroblasts [19]. However, there is still a lack of efficacious treatments for PD at present. In this research, we showed that intratunical injection of BMSCs prevented fibrosis in a rat model of PD, and the effects of BMSCs treatment in the active phase of PD were better than those in the chronic phase. Furthermore, we found that one mechanism of BMSCs treatment was related to increased Smad7 expression in TGF-β1-treated fibroblasts derived from rat penile TA, and there was no effect on cell viability of fibroblasts.

Through DNA microarrays to compare the gene differences between PD plaques and control tunica albugineas, elastase-2B was found to be upregulated in PD plaques [20]. Elastases form a subfamily (elastases 1, 2, 2A, 2B, 3A, and 3B) of serine proteases that hydrolyse many proteins in addition to elastin. Similar to most human elastases, elastase 2B is secreted from the pancreas as a zymogen [21]. However, no studies have confirmed this finding. In this study, increased elastase-2B expression was confirmed in the rats with TGF-β1-induced PD and TGF-β1-induced fibroblasts, and BMSCs injection suppressed elastase 2B expression levels, suggesting that BMSCs might regulate elastase-2B roles in the plaque tissue of PD. Recent studies have confirmed that osteopontin plays an important role in the occurrence and development of pulmonary, liver and renal fibrosis [22–24]. In addition, a study has shown that osteopontin is the direct target of the Smads-mediated TGF-β1 signalling, and that the osteopontin gene promoter can specifically bind to the Smad protein complex and directly participate in TGF-β1/Smad signalling [25]. In our study, increased osteopontin expression levels were found in the rats with PD, and BMSCs injection prevented the osteopontin expression, suggesting that BMSCs might play an important role in regulating osteopontin in the plaque tissue of PD.

Smads are the main components of the TGF-β1 pathway, and the decreased expression of Smad7 in PD plaques may promote fibrosis [2, 26, 27]. Choi et al. [2] found that overexpression of Smad7 could significantly reduce the expression of extracellular matrix proteins such as plasminogen activation inhibitor-1 (PAI-1), fibronectin, collagen I and collagen IV, thus inhibiting the TGF-β1-mediated fibrotic response of fibroblasts derived from human PD plaques. In this study, the Smad7 expression increased after BMSCs treatment in the TA of the rats with PD, indicating that BMSCs injection suppressed fibrosis by regulating Smad7 in the rats with PD. To confirm this hypothesis, we assessed fibroblasts derived from rat penile TA. The fibroblasts were indirectly cocultured with BMSCs and activated by TGF-β1. Moreover, the Smad7 expression vector was used as a positive control. The results showed that the effects of BMSCs were similar to those of the Smad7 expression vector, which blocked the suppression of collagen 3, elastase-2B and osteopontin expression in TGF-β1-treated fibroblasts, indicating that promoting Smad7 expression was a mechanism of BMSCs in the treatment of the PD rat model. Interestingly, the viability of the fibroblasts was not affected by BMSCs, indicating that BMSCs are a possible effective and safe treatment for PD.

However, the antifibrotic of BMSCs should be diverse, but the effects of BMSCs were similar to those of the Smad7 overexpression vector in this study. The possible reason was the different coculture methods: that the fibroblasts were cocultured with BMSCs in a Transwell plate, while they were directly cocultured with the Smad7 overexpression vector. Therefore, multicell coculture systems, such as a three-dimensional environment, need to be studied further. Additionally, the possible mechanisms of increased elastase-2B and osteopontin expression levels in PD plaques are not clear and need to be studied further. Moreover, whether elastase-2B and osteopontin are key factors of BMSCs in the treatment of PD is a continued topic of inquiry.

## Conclusions

Here, the therapeutic effects of BMSCs on a PD rat model at different phases were studied for the first time. Intratunical injection of BMSCs prevented fibrosis in a rat model of PD, and the effects of BMSCs treatment in the active phase of PD were better than those in the chronic phase. Moreover, a possible mechanism of BMSCs treatment was related to increased Smad7 expression in TGF-β1-treated fibroblasts.

## Supplementary Information

**Additional file 1**. The full-length gels of Fig. 5A.

**Additional file 2**. The full-length gels of Fig. 8A.

**Additional file 3**. The full-length gels of Fig. 11A.

## Data Availability

All data in this study are available from the corresponding author upon request.

## Abbreviations

PD: Peyronie’s disease
BMSCs: Bone marrow mesenchymal stem cells
TA: Tunica albuginea
TGF-β1: Transforming growth factor-β1
ADSCs: Adipose tissue-derived stem cells
TIMPs: Metalloproteinases
PM-MSCs: Placental matrix-derived mesenchymal stem cells
PBS: Phosphate-buffered saline
ICP: Intracavernous pressure
CN: Cavernous nerve
HE: Haematoxylin & eosin
