# Supplementary figures and images for: Intratunical injection of rat-derived bone marrow mesenchymal stem cells prevents fibrosis and is associated with increased Smad7 expression in a rat model of Peyronie’s disease
Source: Stem Cell Res Ther. 2022 Jul 30;13:390. doi: 10.1186/s13287-022-03090-w (PMC9338499; doi:10.1186/s13287-022-03090-w)

## The full-length gels of figure 5A

Smad7

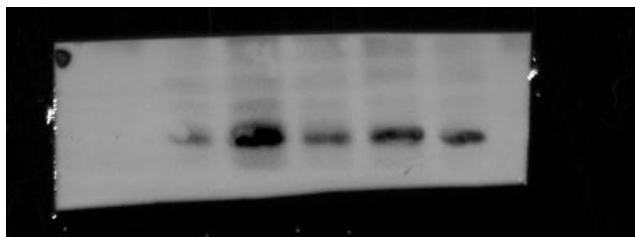

Collagen III

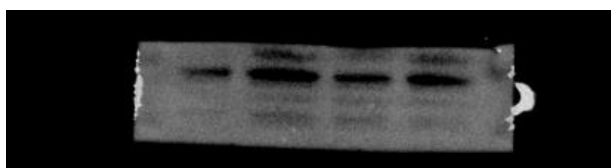

Elastase-IIB

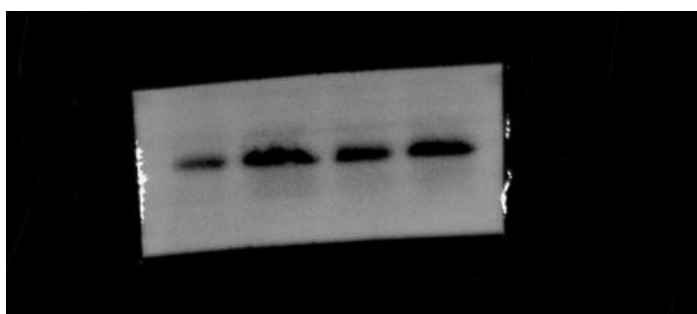

Osteopontin

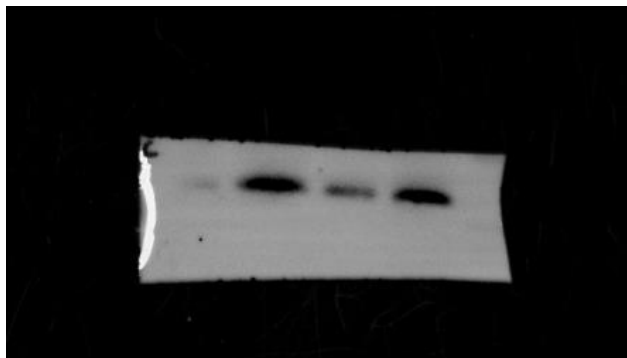

GAPDH

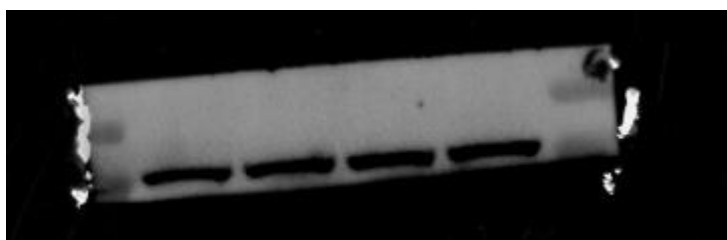

Supplement: Supplementary file 1 — Additional file 1. The full-length gels of Fig. 5A. [file 13287_2022_3090_MOESM1_ESM.pdf]

**The full-length gels of figure 8A**

Smad7

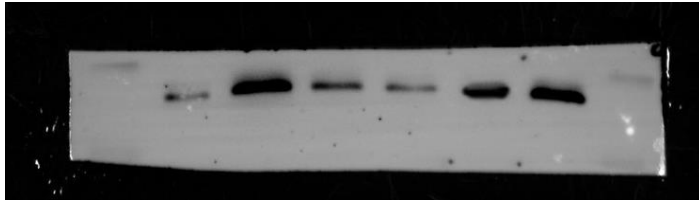

Collagen III

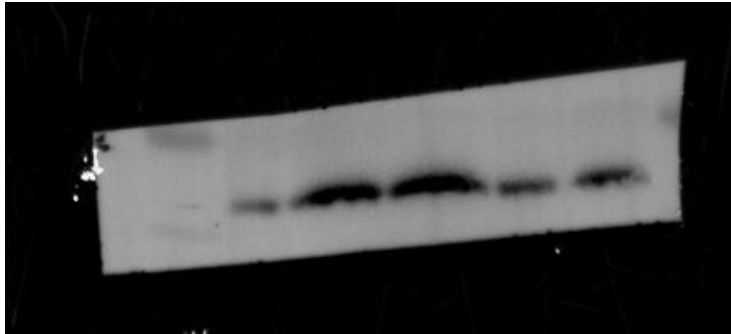

Elastase-IIB

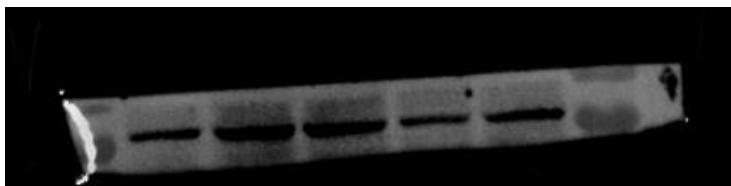

Osteopontin

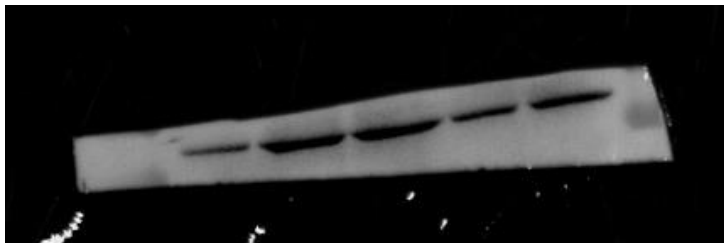

GAPDH

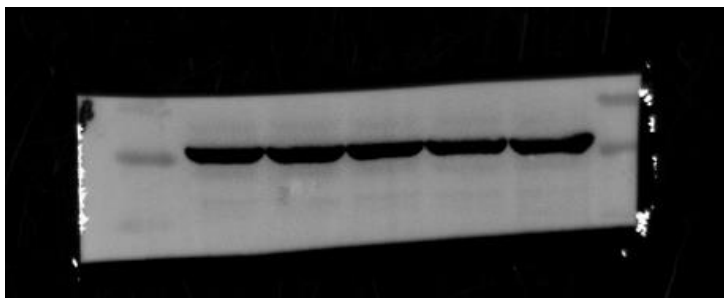

Supplement: Supplementary file 2 — Additional file 2. The full-length gels of Fig. 8A. [file 13287_2022_3090_MOESM2_ESM.pdf]

**The full-length gels of figure 11A.**

Smad7

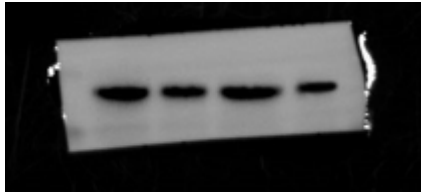

Collagen III

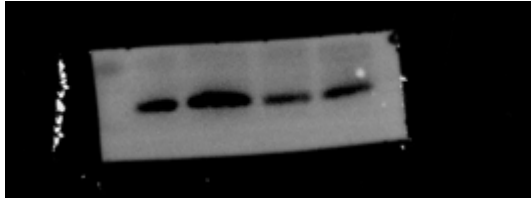

Elastase-IIB

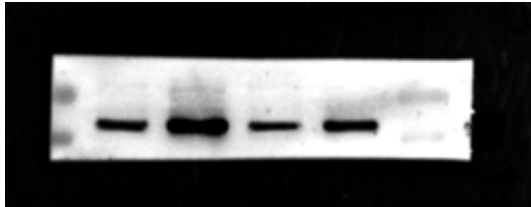

Osteopontin

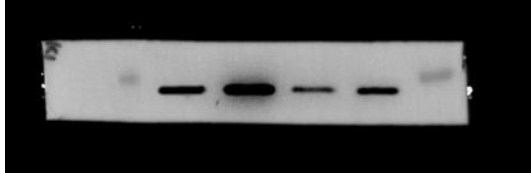

GAPDH

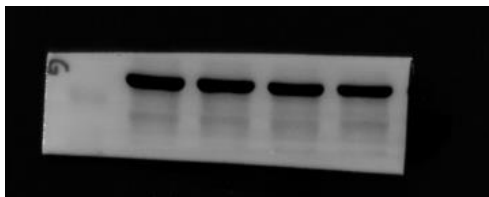

Supplement: Supplementary file 3 — Additional file 3. The full-length gels of Fig. 11A. [file 13287_2022_3090_MOESM3_ESM.pdf]
